# School Connectedness and Risk Behaviors and Experiences Among High School Students — Youth Risk Behavior Survey, United States, 2021

**DOI:** 10.15585/mmwr.su7201a2

**Published:** 2023-04-28

**Authors:** Natalie J. Wilkins, Kathleen H. Krause, Jorge V. Verlenden, Leigh E. Szucs, Emily N. Ussery, Christopher T. Allen, Joi Stinson, Shannon L. Michael, Kathleen A. Ethier

**Affiliations:** ^1^Division of Adolescent and School Health, National Center for HIV, Viral Hepatitis, STD, and TB Prevention, CDC; ^2^Division of Overdose Prevention, National Center for Injury Prevention and Control, CDC; ^3^Division of Violence Prevention, National Center for Injury Prevention and Control, CDC; ^4^Division of Population Health, National Center for Chronic Disease Prevention and Health Promotion, CDC

## Abstract

School connectedness, defined as students’ belief that adults and peers in their school care about their learning as well as about them as persons, has been linked to positive educational, behavioral, and health outcomes in adolescence and into adulthood. Data from the 2021 nationally representative Youth Risk Behavior Survey, conducted during the COVID-19 pandemic, were used to estimate prevalence of students’ perception of school connectedness and examine associations between school connectedness and seven risk behaviors and experiences: poor mental health, marijuana use, prescription opioid misuse, sexual intercourse, unprotected sex, experiencing forced sex, and missing school because of feeling unsafe. Prevalence estimates were generated and pairwise *t*-tests were used to detect differences among student subpopulations by sex, grade, race and ethnicity, and sexual identity; Wald chi-square tests were used to detect differences in risk behaviors by level of connectedness within a subpopulation. Logistic regression models were used to estimate prevalence ratios comparing the prevalence of risk behaviors and experiences of students with high connectedness with students with low connectedness, stratified by demographics. During 2021, 61.5% of U.S. high school students reported feeling connected to others at school. In addition, school connectedness was associated with lower prevalence of every risk behavior and experience examined in this study, although certain associations differed by race and ethnicity and sexual identity (e.g., school connectedness was associated with better mental health outcomes for youths with heterosexual, bisexual, and questioning or other sexual identities, but not for youths who identified as lesbian or gay). These findings can guide public health interventions that promote youth well-being by creating school environments where all youths have a sense of belonging and feel they are cared for and supported.

## Introduction

School connectedness is the sense of being cared for, supported, and belonging, which is fostered by a caring and supportive educational environment and is commonly defined as the “belief by students that adults and peers in the school care about their learning as well as about them as persons (*1*).” School connectedness during adolescence has been linked to positive health outcomes, including reductions in emotional distress, symptoms of poor mental health, and suicidal ideation (*2*,*3*); health risk behaviors (e.g., marijuana and prescription drug misuse) (*3*); and negative experiences (e.g., sexual violence victimization) (*3*), and multiple of these protective effects have been found to last into adulthood (*3*). In addition, school connectedness has been identified as a protective factor for adolescents who might be facing stress, adversity, or marginalization. For example, higher school connectedness has been associated with lower levels of peer victimization, experiences of school violence, and poor mental health among adolescents identifying as lesbian, gay, and bisexual and has been associated with both an increased likelihood of bystander intervention during bullying and an increased likelihood of seeking assistance after being bullied (*4*,*5*). Studies have also found protective associations between school connectedness and adolescent sexual behaviors, including lower prevalence of early sexual debut and lower frequency of sex (*3*).

Understanding the association between adolescents’ perceptions of school connectedness and their behaviors and experiences is important for identifying ways that schools might promote healthy behaviors, protect against risk, and facilitate healthy trajectories. Furthermore, investigating the role of school connectedness as a protective factor for youths across and among racial, ethnic, sexual orientation, and gender identities is necessary for understanding the potential of school connectedness as an intervention (*6*,*7*).

In 2021, for the first time, the Youth Risk Behavior Survey (YRBS) included a single-item measure of school connectedness, providing the opportunity to examine perspectives of connectedness among a nationally representative sample of U.S. high school students. Using YRBS data, this report explores the association between perceptions of school connectedness and adolescent behaviors and experiences. The findings in this report can support the development of interventions and guide decision-making among educational and public health leaders about ways to best promote and protect the health of adolescents.

## Methods

### Data Source

This report includes data from the 2021 YRBS (N = 17,232), a cross-sectional, school-based survey conducted biennially since 1991. Each survey year, CDC collects data from a nationally representative sample of public and private school students in grades 9–12 in the 50 U.S. states and the District of Columbia. Additional information about YRBS sampling, data collection, response rates, and processing is available in the overview report of this supplement (*8*). The prevalence estimates for school connectedness for the overall study population and by sex, race and ethnicity, grade, and sexual identity are available at https://nccd.cdc.gov/youthonline/App/Default.aspx. The full YRBS questionnaire, data sets, and documentation are available at https://www.cdc.gov/healthyyouth/data/yrbs/index.htm. This activity was reviewed by CDC and was conducted consistent with applicable federal law and CDC policy.*

### Measures

This study examined school connectedness and its association with risk behaviors and experiences and demographics. School connectedness was measured as, “Do you agree or disagree that you feel close to people at your school?” with responses coded as high (strongly agree and agree) versus low (not sure, disagree, and strongly disagree) connectedness. Seven risk behaviors and experiences examined were poor mental health, marijuana use, prescription opioid misuse, sexual intercourse, unprotected sex, experiencing forced sex, and missing school because of feeling unsafe (Table 1). Demographic variables included sex (female or male); race and ethnicity (American Indian or Alaska Native [AI/AN], Asian, Black or African American [Black], Hispanic or Latino [Hispanic], Native Hawaiian or other Pacific Islander [NH/OPI], White, or multiracial [selected >1 racial category]; grade (9 and 10 or 11 and 12); and sexual identity (heterosexual, lesbian, gay, bisexual, questioning [I am not sure about my sexual identity/questioning], or other [I describe my identity in some other way] [LGBQ+]). (Persons of Hispanic origin might be of any race but are categorized as Hispanic; all racial groups are non-Hispanic.)

**TABLE 1:** Question and analytic coding for risk behaviors and experiences, by variable assessed — Youth Risk Behavior Survey, United States, 2021

Variable	Question	Response options	Analytic coding
Poor mental health	During the past 30 days, how often was your mental health not good?	Never, rarely, sometimes, most of the time, always	Yes (most of the time, always) versus no (never, rarely, sometimes)
Lifetime marijuana use	During your life, how many times have you used marijuana?	0 times, 1–2 times, 3–9 times, 10–19 times, 20–39 times, 40–99 times, ≥100 times	Yes (1–2 times, 3–9 times, 10–19 times, 20–39 times, 40–99 times, ≥100 times) versus 0 times
Lifetime prescription opioid misuse	During your life, how many times have you taken prescription pain medicine without a doctor's prescription or differently than how a doctor told you to use it?*	0 times, 1–2 times, 3–9 times, 10–19 times, 20–39 times, ≥40 times	Yes (1–2 times, 3–9 times, 10–19 times, 20–39 times, ≥40 times) versus 0 times
Ever sexual intercourse	Have you ever had sexual intercourse?	Yes, no	Yes versus no
No protection at last sexual intercourse	Combination of: (a) The last time you had sexual intercourse, did you or your partner use a condom? (b) The last time you had sexual intercourse with an opposite-sex partner, what one method did you or your partner use to prevent pregnancy? (c) Have you ever had sexual intercourse?	(a) I have never had sexual intercourse, yes, no (b) I have never had sexual intercourse with an opposite-sex partner, no method was used to prevent pregnancy, birth control pills (do not count emergency contraception such as Plan B or the “morning after” pill), condoms, an IUD (such as Mirena or ParaGard) or implant (such as Implanon or Nexplanon), shot (such as Depo-Provera), patch (such as OrthoEvra), or birth control ring (such as NuvaRing), withdrawal or some other method, not sure (c) Yes, no	Yes (Either (a) no, (b) no birth control was used to prevent pregnancy, withdrawal or some other method, not sure, or (c) yes with missing responses to (a) and (b)) versus no (Either (a) I have never had sexual intercourse, yes (b) I have never had sexual intercourse with an opposite-sex partner, birth control pills (do not count emergency contraception such as Plan B or the “morning after” pill), condoms, an IUD (such as Mirena or ParaGard) or implant (such as Implanon or Nexplanon), shot (such as Depo-Provera), patch (such as OrthoEvra), or birth control ring (such as NuvaRing), or (c) No
Ever experienced forced sex	Have you ever been physically forced to have sexual intercourse when you did not want to?	Yes, no	Yes versus no
Missed school because of feeling unsafe	During the past 30 days, on how many days did you not go to school because you felt you would be unsafe at school or on your way to or from school?	0 days, 1 day, 2–3 days, 4–5 days, ≥6 days	Yes (1 day, 2–3 days, 4–5 days, ≥6 days) versus 0 days)

**Abbreviation:** IUD = intrauterine device.

* Instructions for this question specified opioid drugs “For these questions, count drugs such as codeine, Vicodin, OxyContin, Hydrocodone, and Percocet.” However, if students considered nonopioid prescription pain medications when answering this question, an overestimation of prescription opioid misuse prevalence might have occurred.

### Analysis

Prevalence estimates of high connectedness among all students and stratified by demographic category were calculated, and pairwise *t*-tests with Taylor series linearization were conducted to detect differences within categories. The prevalence of seven risk behaviors and experiences (Table 1) were estimated among students overall and stratified by demographic category, with estimates for students with high and low connectedness. Pairwise *t*-tests were used to detect prevalence differences in connectedness (high and low), stratified by demographic characteristic; Wald chi-square tests were used to detect prevalence differences in risk behaviors by level of connectedness within a demographic stratum. Finally, unadjusted logistic regression models with a statement for predicted marginal proportions were used to estimate prevalence ratios (PRs) of each risk behavior among students with high connectedness compared with students with low connectedness. Analyses were conducted in SAS-callable SUDAAN (version 11.0.3; RTI International) by using sample weights to account for complex survey design and nonresponse. Estimates were considered statistically significant if the 95% CI did not include 1.0 or p<0.05. Prevalence estimates with a denominator <30 were considered statistically unreliable and therefore were suppressed (*8*).

## Results

### School Connectedness Overall and by Population Characteristics

During 2021, 61.5% of U.S. high school students reported that they felt connected to others at school (Table 2). Prevalence of feeling connected to others at school was highest among male (65.5%), Asian (66.7%), 9th- and 10th-grade (63.3.%), and heterosexual (65.1%) students. The lowest prevalence of feeling connected to others at school was reported among students who were female (57.6%), AI/AN (53.9%) or Black (53.9%), in 11th and 12th grade (59.8%) and had questioning or other sexual identities (48.3%).

**TABLE 2:** Prevalence of school connectedness by demographic characteristics — Youth Risk Behavior Survey, United States, 2021*

Characteristic	Felt connected to others at school^†^ % (95%CI)
**Sex^†,§^**	
Female	57.6 (54.3–60.9)
Male	65.5 (63.4–67.6)
**Race and ethnicity^§,¶^**	
American Indian or Alaska Native	53.9 (43.4–64.1)
Asian	66.7 (56.6–75.5)
Black or African American	53.9 (50.2–57.6)
Native Hawaiian or other Pacific Islander	60.3 (51.3–68.6)
White	65.2 (62.5–67.8)
Hispanic or Latino	57.5 (55.1–59.9)
Multiracial	59.8 (55.7–63.7)
**Grade**	
9 and 10	63.3 (60.6–65.9)
11 and 12	59.8 (56.1–63.4)
**Sexual identity^§^**	
Heterosexual	65.1 (62.3–67.8)
Lesbian or gay	55.0 (46.3–63.5)
Bisexual	54.2 (49.1–59.2)
Questioning or other	48.3 (43.8–52.9)
**Total**	**61.5 (59.0–63.9)**

* N = 17,232 respondents. Because the state and local questionnaires differ by jurisdiction, students in these schools were not asked all national YRBS questions. Therefore, the total number (N) of students answering each question varied. Percentages in each category are calculated on the known data.

^†^ On the basis of the answer (“strongly agree” or “agree” [not sure, disagree, strongly disagree]) to the survey question, “Do you agree or disagree that you feel close to people at your school?”

^§^ On the basis of *t*-tests with Taylor series linearization (p<0.05), statistically significant differences were observed between the following subgroups of students: female versus male; American Indian or Alaska Native versus White; Asian versus Black or African American (Black); Black versus multiracial; Black versus White; Hispanic or Latino (Hispanic) versus White; multiracial versus White; lesbian or gay versus heterosexual; bisexual versus heterosexual; questioning or other versus heterosexual; bisexual versus questioning or other.

^¶^ Persons of Hispanic origin might be of any race but are categorized as Hispanic; all racial groups are non-Hispanic.

### School Connectedness and Risk Behaviors and Experiences

Students who reported feeling connected to others at school had lower prevalence of all risk behaviors and experiences compared with students who reported not feeling connected to others at school (Table 3). These observations included lower prevalence of poor mental health (22.0% versus 40.1%), lifetime marijuana use (25.8% versus 32.6%), lifetime prescription opioid misuse (9.6% versus 16.8%), sexual intercourse (27.6% versus 34.9%), unprotected sex (7.9% versus 12.7%), experiencing forced sex (6.6% versus 12.1%), and missing school because of feeling unsafe (5.9% versus 11.0%). The association between high connectedness and lower risk behaviors and experiences was consistent for both male and female students and across all grades except for sexual intercourse, which was not different among 11th- and 12th-grade students reporting high versus low connectedness.

**TABLE 3:** Prevalence of risk behavior by level of school connectedness and demographic characteristics — Youth Risk Behavior Survey, United States, 2021*

Characteristic	Level of school connectedness^†^	Mental health not good in past 30 days % (95% CI)	Lifetime marijuana use % (95% CI)	Lifetime prescription opioid misuse % (95% CI)	Ever sexual intercourse % (95% CI)	Unprotected sex % (95% CI)	Ever experienced forced sex % (95% CI)	Skipped school in past 30 days because felt unsafe % (95% CI)
Total	High	22.0 (20.5–23.6)^§^	25.8 (23.4–28.3)^§^	9.6 (8.6–10.7)^§^	27.6 (25.1–30.1)^§^	7.9 (6.8–9.1)^§^	6.6 (5.4–7.9)^§^	5.9 (4.9–7.1)^§^
	Low	40.1 (38.0–42.3)	32.6 (29.0–36.4)	16.8 (14.9–18.8)	34.9 (32.1–37.8)	12.7 (11.0–14.7)	12.1 (11.2–13.0)	11.0 (9.3–13.0)
**Sex**								
Female	High	32.7 (29.6–35.9)^§^	28.3 (25.5–31.2)^§^	12.2 (10.6–13.9)^§^	27.3 (24.3–30.6)^§^	9.3 (8.0–10.7)^§^	11.2 (9.4–13.3)^§^	7.1 (5.61–8.9)^§^
	Low	51.2 (49.1–53.2)	35.0 (30.4–39.9)	19.3 (16.8–22.1)	35.8 (32.4–39.4)	12.5 (10.1–15.5)	17.8 (16.1–19.5)	13.5 (11.5–15.9)
Male	High	13.1 (11.4–15.1)^§^	23.6 (21.2–26.3)^§^	7.2 (6.28–8.3)^§^	27.8 (25.0–30.8)^§^	6.8 (5.5–8.3)^§^	2.8 (1.9–4.1)^§^	4.8 (3.9–6.1)^§^
	Low	26.9 (23.9–30.1)	29.5 (26.3–32.9)	13.3 (11.5–15.4)	33.5 (30.2–37.0)	12.7 (10.8–14.9)	4.9 (4.0–5.9)	8.0 (6.2–10.3)
**Race and ethnicity^¶^**								
American Indian or Alaska Native	High	19.7 (10.1–35.0)^§^	16.5 (7.3–33.0)	11.8 (3.4–34.0)	27.7 (12.0–51.9)	4.8 (1.6–13.7)	13.8 (4.9–33.0)	7.6 (2.9–18.4)^§^
	Low	44.6 (31.0–59.0)	39.9 (22.6–60.2)	19.4 (10.5–33.2)	43.2 (27.8–60.1)	—**	22.5 (10.9–40.7)	22.7 (11.0–41.1)
Asian	High	16.9 (12.5–22.6)^§^	8.9 (5.6–13.8)	8.6 (5.8–12.5)^§^	9.4 (6.9–12.6)	2.5 (0.9–6.6)	2.2 (1.0–4.6)^§^	2.3 (1.2–4.1)^§^
	Low	33.7 (28.9–38.8)	12.8 (9.9–16.3)	16.1 (11.0–22.9)	13.8 (10.9–17.3)	6.0 (3.6–9.9)	8.3 (6.0–11.5)	7.8 (5.0–12.0)
Black or African American	High	20.1 (17.1–23.4)^§^	34.2 (26.1–43.5)	10.7 (7.8–14.6)^§^	36.7 (30.8–43.1)	12.1 (9.0–15.9)	5.3 (3.6–7.6)^§^	9.1 (6.3–12.9)^§^
	Low	32.9 (28.0–38.2)	33.7 (26.4–41.9)	17.8 (13.9–22.5)	35.6 (28.0–44.0)	15.1 (11.2–20.0)	10.3 (7.4–14.1)	14.3 (10.1–19.9)
Native Hawaiian or other Pacific Islander	High	—	—	—	—	—	—	—
	Low	—	—	—	—	—	—	—
White	High	21.9 (19.5–24.5)^§^	24.1 (22.2–26.1)^§^	8.8 (7.1–1.0)^§^	27.5 (24.9–30.4)^§^	6.4 (5.1–8.0)^§^	6.4 (5.2–7.9)^§^	4.4 (3.1–6.2)^§^
	Low	43.8 (41.0–46.6)	31.4 (28.3–34.6)	15.9 (13.3–19.0)	36.1 (33.3–39.0)	12.1 (10.1–14.4)	12.5 (10.9–14.2)	9.6 (7.6–12.0)
Hispanic or Latino	High	23.4 (21.8–25.2)^§^	29.3 (27.1–31.6)	11.3 (9.2–13.8)^§^	28.2 (25.7–30.9)^§^	11.2 (9.3–13.3)^§^	8.1 (6.8–9.6)^§^	8.8 (6.2–12.4)^§^
	Low	38.9 (35.0–43.1)	35.7 (28.0–44.3)	18.3 (15.0–22.1)	36.6 (32.0–41.5)	14.2 (11.9–16.8)	12.7 (10.3–15.6)	11.9 (9.4–14.8)
Multiracial	High	27.4 (22.4–33.1)^§^	34.5 (24.0–46.9)	10.4 (6.9–15.4)	31.7 (24.5–39.9)	8.1 (5.1–12.5)	10.4 (7.3–14.7)	5.1 (2.6–10.0)^§^
	Low	43.0 (35.1–51.3)	41.7 (32.2–51.8)	15.1 (10.0–22.1)	37.9 (31.5–44.7)	13.4 (8.9–19.6)	13.4 (9.3–19.0)	11.2 (8.1–15.3)
**Grade**								
9 and 10	High	21.4 (19.0–24.0)^§^	18.1 (15.2–21.4)^§^	10.2 (8.9–11.8)^§^	16.9 (14.3–19.8)^§^	5.5 (4.3–7.0)^§^	6.2 (5.0–7.7)^§^	6.7 (5.1–8.7)^§^
	Low	39 (35.6–42.4)	23.7 (20.6–27.2)	17.1 (15.1–19.4)	24.1 (21.2–27.3)	9.4 (7.8–11.2)	11.0 (9.6–12.6)	11.4 (9.5–13.7)
11 and 12	High	22.5 (20.9–24.2)^§^	34.1 (31.4–36.9)^§^	8.9 (7.8–10.2)^§^	39.1 (35.9–42.4)	11.0 (9.2–13.2)^§^	7.0 (5.6–8.7)^§^	4.8 (3.9–5.8)^§^
	Low	41.4 (38.8–44.0)	40.5 (36.2–45.0)	16.1 (13.8–18.8)	44.6 (39.3–49.9)	16.2 (13.5–19.3)	12.8 (11.2–14.5)	10.2 (8.1–12.9)
**Sexual identity**								
Heterosexual	High	16.4 (15.3–17.6)^§^	24.2 (22.4–26.1)^§^	7.3 (6.4–8.3)^§^	27.0 (24.5–29.6)	7.0 (5.9–8.2)^§^	3.8 (2.8–5.1)^§^	4.6 (3.6–5.9)^§^
	Low	31.3 (28.4–34.4)	31.1 (27.7–34.7)	13.0 (10.8–15.6)	34.7 (31.4–38.2)	12.5 (10.7–14.5)	7.1 (6.1–8.2)	8.5 (7.0–10.4)
Lesbian or gay	High	35.2 (27.5–43.8)	28.5 (17.4–43.1)	11.5 (7.2–17.8)^§^	29.2 (19.5–41.1)	6.2 (2.6–14.2)	12.7 (7.4–20.8)^§^	12.7 (6.1–24.6)
	Low	50.2 (37.1–63.3)	33.3 (26.8–40.6)	30.1 (22.1–39.6)	35.3 (23.9–48.7)	13.4 (6.6–25.2)	21.9 (16.4–28.6)	15.0 (9.4–23.3)
Bisexual	High	45.8 (40.1–51.5)^§^	41.3 (31.1–52.2)	19.9 (16.9–23.2)^§^	36.9 (32.3–41.8)	16.6 (12.6–21.5)	20.0 (16.2–24.5)^§^	9.7 (6.8–13.5)^§^
	Low	63.8 (59.3–68.1)	46.4 (39.4–53.6)	27.5 (23.1–32.3)	44.7 (40.0–49.4)	16.4 (12.5–21.2)	28.1 (24.4–32.1)	16.7 (12.5–22.0)
Questioning or other	High	43.2 (35.9–50.9)^§^	24 (18.6–30.4)	17.4 (13.0–23.0)	22.6 (16.2–30.6)	6.3 (3.7–10.6)	13.6 (8.6–20.9)	8.5 (4.7–15.1)^§^
	Low	60.1 (55.9–64.2)	29.6 (24.8–34.9)	21.5 (17.9–25.5)	25.7 (22.5–29.3)	11.6 (8.5–15.7)	19.6 (16.6–22.9)	16.1 (12.6–20.4)

* N = 17,232 respondents. Because the state and local questionnaires differ by jurisdiction, students in these schools were not asked all national YRBS questions. Therefore, the total number (N) of students answering each question varied. Percentages in each category are calculated on the known data.

^†^ In answer to the question, “Do you agree or disagree that you feel close to people at your school,” “High” = Strongly agree, agree; “Low” = Not sure, disagree, strongly disagree.

^§^ Wald chi-square test indicates statistically significant difference (p<0.05) for students who reported high versus low level of school connectedness.

^¶^ Persons of Hispanic or Latino (Hispanic) origin might be of any race but are categorized as Hispanic; all racial groups are non-Hispanic.

** Prevalence estimates with a denominator <30 were considered statistically unreliable and therefore were suppressed.

### School Connectedness and Risk Behaviors and Experiences by Racial and Ethnic Identity

Across all racial and ethnic identities, students who reported high levels of school connectedness also reported lower prevalence of poor mental health compared with students who reported low school connectedness (AI/AN: 19.7% versus 44.6%; Asian: 16.9% versus 33.7%; Black: 20.1% versus 32.9%; Hispanic: 23.4% versus 38.9%; White: 21.9% versus 43.8%; and multiracial: 27.4% versus 43.0%) (Table 3). Among Asian (PR = 0.26) and Black (PR = 0.51) students, school connectedness had the strongest association with lower prevalence of ever experiencing forced sex (Table 4). Among Hispanic students, school connectedness was most strongly associated with lower prevalence of poor mental health (PR = 0.60). Among multiracial students, school connectedness was associated only with lower prevalence of poor mental health (PR = 0.64); among White students, school connectedness was most strongly associated with lower prevalence of missing school because of feeling unsafe (PR = 0.46).

**TABLE 4:** Prevalence ratios comparing risk behaviors among students with low and high school connectedness — Youth Risk Behavior Survey, United States, 2021*

Characteristic	Mental health not good in past 30 days PR (95% CI)	Lifetime marijuana use PR (95% CI)	Lifetime prescription opioid misuse PR (95% CI)	Ever sexual intercourse PR (95% CI)	Unprotected sex PR (95% CI)	Ever experienced forced sex PR (95% CI)	Skipped school in past 30 days because felt unsafe PR (95% CI)
**Total**	**0.55 (0.51–0.59)^†^**	**0.79 (0.72–0.87)^†^**	**0.57 (0.49–0.67)^†^**	**0.79 (0.71–0.88)^†^**	**0.62 (0.53–0.73)^†^**	**0.54 (0.45–0.65)^†^**	**0.54 (0.44–0.66)^†^**
**Sex**							
Female	0.64 (0.59–0.70)^†^	0.81 (0.71–0.92)^†^	0.63 (0.53–0.75)^†^	0.76 (0.66–0.88)^†^	0.74 (0.58–0.94)^†^	0.63 (0.52–0.77)^†^	0.52 (0.41–0.67)^†^
Male	0.49 (0.39–0.61)^†^	0.80 (0.72–0.89)^†^	0.54 (0.45–0.66)^†^	0.83 (0.73–0.94)^†^	0.53 (0.41–0.69)^†^	0.57 (0.38–0.86)^†^	0.60 (0.42–0.85)^†^
**Race and ethnicity** ^§^							
American Indian or Alaska Native	0.44 (0.23–0.84)^†^	0.41 (0.17–1.01)	0.61 (0.17–2.21)	0.64 (0.25–1.66)	—^¶^	0.61 (0.16–2.37)	0.33 (0.11–1.05)
Asian	0.50 (0.39–0.65)^†^	0.69 (0.41–1.17)	0.54 (0.31–0.93)^†^	0.68 (0.46–1.01)	0.41 (0.11–1.50)	0.26 (0.11–0.61)^†^	0.29 (0.12–0.69)^†^
Black or African American	0.61 (0.5–0.73)^†^	1.02 (0.75–1.39)	0.60 (0.41–0.89)^†^	1.03 (0.83–1.28)	0.80 (0.52–1.22)	0.51 (0.31–0.83)^†^	0.64 (0.48–0.85)^†^
Native Hawaiian or other Pacific Islander	—	—	—	—	—	—	—
White	0.50 (0.46–0.54)^†^	0.77 (0.69–0.85)^†^	0.55 (0.41–0.76)^†^	0.76 (0.67–0.86)^†^	0.53 (0.40–0.68)^†^	0.51 (0.41–0.65)^†^	0.46 (0.33–0.65)^†^
Hispanic or Latino	0.60 (0.54–0.67)^†^	0.82 (0.66–1.02)	0.62 (0.49–0.77)^†^	0.77 (0.69–0.86)^†^	0.79 (0.66–0.93)^†^	0.64 (0.51–0.80)^†^	0.75 (0.53–1.04)
Multiracial	0.64 (0.47–0.87)^†^	0.83 (0.61–1.12)	0.69 (0.39–1.21)	0.84 (0.63–1.11)	0.60 (0.34–1.07)	0.78 (0.44–1.38)	0.46 (0.20–1.06)
**Grade**							
9 and 10	0.55 (0.49–0.62)^†^	0.76 (0.65–0.88)^†^	0.60 (0.50–0.71)^†^	0.70 (0.55–0.88)^†^	0.58 (0.44–0.77)^†^	0.56 (0.47–0.68)^†^	0.59 (0.43–0.79)^†^
11 and 12	0.54 (0.49–0.60)^†^	0.84 (0.75–0.94)^†^	0.55 (0.46–0.66)^†^	0.88 (0.76–1.01)	0.68 (0.53–0.89)^†^	0.55 (0.43–0.70)^†^	0.47 (0.37–0.59)^†^
**Sexual identity**							
Heterosexual	0.52 (0.47–0.59)^†^	0.78 (0.70–0.86)^†^	0.56 (0.45–0.70)^†^	0.78 (0.69–0.88)^†^	0.56 (0.46–0.68)^†^	0.53 (0.38–0.75)^†^	0.54 (0.40–0.73)^†^
Lesbian or gay	0.70 (0.46–1.07)	0.86 (0.56–1.31)	0.38 (0.23–0.62)^†^	0.83 (0.52–.31)	0.46 (0.11–1.97)	0.58 (0.34–0.99)^†^	0.85 (0.35–2.04)
Bisexual	0.72 (0.64–0.81)^†^	0.89 (0.72–1.1)	0.72 (0.59–0.88)^†^	0.83 (0.68–1.00)	1.01 (0.74–1.39)	0.71 (0.57–0.89)^†^	0.58 (0.34–0.97)^†^
Questioning or other	0.72 (0.61–0.85)^†^	0.81 (0.64–1.04)	0.81 (0.62–1.06)	0.88 (0.63–1.23)	0.54 (0.28–1.05)	0.69 (0.45–1.08)	0.53 (0.26–1.08)

**Abbreviation:** PR = prevalence ratio.

* N = 17,232 respondents. Because the state and local questionnaires differ by jurisdiction, students in these schools were not asked all national YRBS questions. Therefore, the total number (N) of students answering each question varied. Percentages in each category are calculated on the known data.

^†^ 95% CI did not cross null value (1.0).

^§^ Persons of Hispanic or Latino (Hispanic) origin might be of any race but are categorized as Hispanic; all racial groups are non-Hispanic.

^¶^ Prevalence estimates with a denominator <30 were considered statistically unreliable and therefore were suppressed.

### School Connectedness and Risk Behaviors and Experiences by Sexual Identity

School connectedness was associated with lower prevalence of poor mental health among students who identified as heterosexual (16.4% versus 31.3%), bisexual (45.8% versus 63.8%), or questioning or other (43.2% versus 60.1%), but not among students who identified as lesbian or gay (Table 3). Among heterosexual students and students with questioning or other sexual identities, school connectedness was most strongly associated with lower prevalence of poor mental health (PR = 0.52) and (PR = 0.72), respectively (Table 4). Among lesbian or gay students, school connectedness was most strongly associated with lower prevalence of lifetime prescription opioid misuse (PR = 0.38). Among bisexual students, school connectedness was most strongly associated with lower prevalence of missing school because of feeling unsafe (PR = 0.58).

## Discussion

This report provides the first national prevalence estimates of school connectedness among U.S. high school students stratified by sex, race and ethnicity, grade, and sexual identity and examines the associations between school connectedness and a range of youth risk behaviors and experiences. Previous research links school connectedness with fewer risk behaviors and adverse experiences among adolescents and indicates that this protective effect might improve the health trajectories of adolescents into adulthood (*3*,*9*). Findings from the current study illustrate that during 2021, approximately one half of all U.S. high school students (61.5%) reported feeling connected to others at school. This pattern held for all student subpopulations stratified by demographics, except for students with questioning or other sexual identities (48.3%). However, prevalence of school connectedness was found to vary by race and ethnicity and sexual identity. School connectedness was highest among Asian students and lower among AI/AN, Black, Hispanic, and multiracial students compared with their White peers. School connectedness was also lower among students who identify as lesbian or gay, bisexual, and questioning or other compared with their peers who identify as heterosexual. These data were collected during the COVID-19 pandemic, and while the effect of the pandemic is unknown, findings are consistent with previous research indicating that prevalence of connectedness is lowest among youths who have experienced racism at school (*10*); identify as LGBQ+ (*11*); and are multiply marginalized and underrepresented (i.e., youths who hold minority racial and ethnic and sexual identities) (*7*). Creating school environments that intentionally focus on students with marginalized identities by proactively addressing discrimination and fostering inclusivity supports positive health and development for all students and might be an important mechanism by which to eliminate inequities in school connectedness (*12*).

Overall, school connectedness was associated with lower prevalence of every risk behavior and experience examined in this study. School connectedness was associated with better mental health during the past 30 days among high school students overall and among all student subpopulations, except among students who identify as lesbian or gay. Robust evidence has demonstrated that school communities can positively influence student mental health, including fostering emotional resilience and lessening emotional distress, anxiety, and depression (*2*,*3*). Longitudinal studies have also found causal associations between school connectedness in adolescence and emotional well-being in adulthood (*3*). Similar to findings in this report, previous studies have indicated that sexual and gender minority youths describe school climate as less positive and report less connection with adults at school, which might contribute to lower connectedness overall and compromise the potential for connectedness to serve a protective role (*6*).

For substance use outcomes, school connectedness was associated with a lower prevalence of lifetime prescription opioid misuse overall and across a majority of subpopulations by sex, grade, race and ethnicity, and sexual identity with three exceptions: there was no association among AI/AN students, multiracial students, or students with questioning or other sexual identities. School connectedness was also associated with a lower prevalence of lifetime marijuana use overall, across sex and grade levels, and among White and heterosexual students. These overall findings align with previous research demonstrating a protective association between school connectedness and substance use (*9*). However, findings from the subgroup analyses indicate the association with lifetime marijuana use might not exist among all subpopulations, including youths from racial and ethnic or sexual minority groups. Among sexual minority youths, one previous study found no association between school connectedness and lifetime marijuana use (*13*), whereas another observed a significant negative association with current (e.g., past 30 days) marijuana use (*14*). Thus, the lack of associations in the current study might partially be a result of the lifetime marijuana use measure, which includes youths who do not currently use marijuana, or it might demonstrate that school connectedness is not a strong correlate of marijuana use among certain subpopulations.

For violence outcomes, school connectedness was associated with lower prevalence of ever experiencing forced sex among all youth subpopulations, except among AI/AN or multiracial youths and those with questioning or other sexual identities; limited sample sizes and wide CIs might explain findings that were not statistically significant among these groups. Youths who have experienced sexual violence trauma often report feelings of isolation and distrust, which could impede their sense of connection and belonging in school (*15*). School connectedness was also associated with lower prevalence of skipping school because of feeling unsafe among students across sex and grade; among Asian, Black, White students; and among heterosexual or bisexual students. School safety reflects an aspect of school community and climate that facilitates connectedness. Perceptions of safety might indicate supportive school environments where students are less likely to experience violence, victimization, and punitive discipline and thus influence students’ feelings of connectedness to school, including among students with identities that are often marginalized, such as LGBQ+, Black, and Hispanic youths (*11*).

For sexual risk outcomes, school connectedness among Hispanic, White, and heterosexual students was associated with lower prevalence of both ever having sex and having unprotected sex at last sexual intercourse. School connectedness was associated with lower prevalence of unprotected sex across sex and grade and lower prevalence of ever having sex among males and 9th- and 10th-grade students. Previous research has highlighted the potential of school connectedness as a protective factor for adolescent sexual health. A recent systematic review demonstrated protective associations between school connectedness and ever having sex, early sexual debut, frequency of sex, and condom and contraceptive use among adolescents (*16*). In this study, protective effects of school connectedness on sexual activity were only observed among younger students. Prevalence of sexual behaviors increases as students age (https://yrbs-explorer.services.cdc.gov/#/), which provides important context when interpreting null associations between school connectedness and ever having sex among 11th and 12th grade students. Future studies should investigate how social connectedness with peers and romantic partners could affect sexual behaviors over time (*16*).

The COVID-19 pandemic caused widespread disruptions to school operations during the time when these data were collected and increased stress and trauma for certain youths and their families (*17*). Although findings indicate consistent associations between students feeling connected to others at school and lower levels of risk behaviors and experiences, data from this study are cross-sectional, and causal direction cannot be inferred. These findings indicate that school connectedness might have a protective or buffering effect, reducing students’ risk behaviors and experiences in the context of a pandemic and increased adversity. In addition, engaging in risk behaviors or experiencing risk might inhibit students’ ability to feel connected to others in their school.

Schools can play a critical role in promoting students’ health and development by creating environments where all students feel that they are cared for, supported, and belong (*6*). Establishing safe and supportive schools for adolescents involves creating an antidiscriminatory environment, which includes layers of protection for students by building caring relationships between students and teachers, managing classrooms effectively, encouraging family engagement, and offering staff wellness and professional development (*6*). School connectedness initiatives that foster inclusion and apply culturally informed practices might more effectively foster positive student health outcomes for all students by engaging students who are more likely to experience poor mental health and risk behaviors (*6*,*18*). School partnerships with community-based health services providers might enhance the ability of schools to meet the needs of student populations at high risk for negative health outcomes. Finally, encouraging students to participate in efforts to enhance school climate and offering positive youth engagement opportunities with community partners has the potential to increase student engagement and foster connectedness (*6*).

## Limitations

General limitations for the YRBS are available in the overview report of this supplement (*8*). The findings in this report are subject to at least four additional limitations. First, the data used in these analyses are cross-sectional and provide a single point-in-time estimate for all variables; therefore, causality and direction of associations between school connectedness and student behaviors and experiences cannot be inferred. Second, the multidimensional characteristics of connectedness, including perceptions of relationships among adults, peers, and the broader school environment, might not be captured by the single item used to measure school connectedness in this study. Third, the limited cell sizes in certain stratified analyses resulted in data suppression for certain racial and ethnic groups. Other racial and ethnic groups have imprecise CIs and might be subject to type II error of failing to reject a false null hypothesis with the Wald chi-square tests. Finally, student responses might only reflect connectedness at a particular point of time; therefore, prevalence of connectedness could vary over time.

## Future Directions

This study aligns with previous research signaling the potential of school connectedness to serve as a protective factor for certain students. However, more research is needed to understand intersecting factors that might contribute to students’ sense of connectedness to the school environment. A variety of strategies exist that schools can use to improve school connectedness; CDC currently recommends strategies such as classroom management, youth development programs that engage students in community settings and bring mentors into schools, and improving LGBQ+ inclusivity (https://www.cdc.gov/healthyyouth/protective/school-connectedness/connectedness_schools.htm). CDC’s Technical Package on Youth Violence Prevention (https://www.cdc.gov/violenceprevention/pdf/yv-technicalpackage.pdf) also highlights the best available evidence for programs and policies to reduce violence, including school-based programs. Schools likely vary in their ability and inclination to put in place these strategies and others like them (e.g., social-emotional learning approaches that teach skills to support students’ social and emotional development). Additional research to understand the interplay of school strategies and students’ beliefs about school connectedness can help set direction for school implementation.

Because of differences in the experience of school connectedness by race and ethnicity and sexual identity, deficits in this important protective factor have long-term implications for students’ health and well-being into adulthood (*3*). More research is needed to identify and dismantle social and structural barriers to improving school connectedness among young persons from racial and ethnic minority groups and LGBQ+ students. Data from CDC’s Adolescent Behaviors and Experiences Survey found that Asian, Black, and multiracial students were most likely to experience racism in school; even among those who reported feeling connected to others at school, the majority had experienced racism (*10*). Implementing policies and practices that prevent and address racism at school might improve the school environment and students’ feelings of connectedness (*18*). Strategies that improve school environments for LGBQ+ students are well established and include school policies and practices such as having student-led clubs (e.g., Gender and Sexualities Alliances [GSAs]), enforced antiharassment policies, identified safe spaces for students, and professional development for school staff on the importance of inclusivity. These strategies create school environments that benefit all students and have been linked to improved health and development outcomes for both LGBQ+ students and their heterosexual peers (*12*). Recent data from CDC’s School Health Profiles survey indicate that, although approximately all schools prohibit harassment of LGBQ+ students and 80% identify safe spaces, only 44% of secondary schools have GSAs and 30% provide training to teachers and school staff on supporting LGBQ+ students (https://www.cdc.gov/healthyyouth/data/profiles/pdf/2020/CDC-Profiles-2020.pdf).

Because of the broad and robust association between school connectedness and the behaviors and experiences of U.S. high school students, it is critical to identify individual, social, structural, and environmental factors that serve as barriers to connectedness and continue to investigate what is needed to effectively create safe and supportive school environments that foster connection.

## Conclusion

During 2021, approximately one half of U.S. high school students overall and across sex, race and ethnicity, grade, and a majority of sexual identities reported a high level of connectedness to school; racial and ethnic and sexual minority students reported lower levels of school connectedness than their White and heterosexual peers. Moreover, this study found that school connectedness was associated with a lower prevalence of all health risk behaviors and experiences, and the association between school connectedness and certain health risk behaviors and experiences varied across racial and ethnic groups and sexual identities. These findings align with previous cross-sectional and longitudinal research linking school connectedness to better health outcomes for youths (*9*) and highlight the importance of school-based strategies that strengthen school connectedness and protect against multiple adolescent health risks. School programs and practices that promote safe and supportive environments and foster inclusion (e.g., GSAs, multicultural groups, and inclusivity training for staff members) might play an important role in improving school connectedness among all youths, including racial and ethnic and sexual identity minority adolescents (*6*,*18*).

## Footnotes

* 45 C.F.R. part 46.102(l)(2), 21 C.F.R. part 56; 42 U.S.C. §241(d); 5 U.S.C. §552a; 44 U.S.C. §3501 et seq.
